# What of the Harvest?

**Published:** 1905-06

**Authors:** 


### Editorial.

### WHAT OF THE HARVEST?

Not the harvest of the fertile fields; not the harvest of souls that our evangelist is seeking to ingather to the fold; not the harvest of gold that the financier hopes to accumulate to make old age a peaceful decline into the valley of silence; not any or all of these, but a harvest of professional power, granaries, not of grain, but of practical skill, stored up for the future welfare of the individual and the world.

We have reached, in the years 1904–05, the close of that session, ending three years, which the dental colleges of this country at St. Louis decided was amply sufficient to make a dentist. The three years have been completed, and before the expiration of the month of June all the dental schools from New England to California will have sent out their quota of senior scholars to battle with the conflicting elements, which are ever waging a war against the new man who ventures to meet their antagonizing influences.

Over this broad land there is a small army of earnest and conscientious teachers in dentistry, who have had to contend between their better natures and the sordid elements that make up the two forces in every individual life, and are now, it is presumed, asking the ever-recurring question, Have I succeeded in imparting a portion of my knowledge and skill to the aspiring youth, that his work may be a credit to himself and to his profession? The man who fails to feel this anxiety as to results at the close of every session seems to the writer to have failed to develop the finer sense of the teacher, a feeling of not having approached his ideal. The individual without an ideal to which he may continually aspire is, from the inception of his work, a failure. Teaching is something more than a mere repetition of the dry bones of facts. It must have within these the spirit of the teacher, to give these life and power, and without this they are dead material incapable of furnishing the pabulum necessary to make the fully developed man.

The spring of 1905 finds us, as teachers of dentistry, face to face with the problem of development. The entire dental teaching world is, and has been for several years, endeavoring to solve the educational problem. The man of one idea has solved this for himself. He may be found on the side of the highest preliminary education possible as a panacea for all our educational woes. Again, he will rest content only in a thorough medical education, and that alone will suffice. Again, the man with one idea will have the whole foundation of dental education based on ability to make a denture thoroughly and artistically. The man of broad ideas has been endeavoring for years to harmonize all of these into one composite whole, in which no one will have a preponderance, but each will be given due weight and in proper order of acquirement. It is a healthy sign of growth, with corresponding vigor, that all over the dental world this question as to the best methods of educating dental students is, and has been, an absorbing topic. The writer is not aware that in any other profession is this regarded with the same interest or of equal importance. It will be a subject of continued interest in the Fédération Dentaire Internationale to meet at Hanover, Germany, in August. It will doubtless have a place in the Councils of the British Dental Association, and naturally should be a leading topic in the Association of Faculties of this country. That there will be unanimity of opinion is not to be expected. The one idea man prevails in Europe, and the broad man has yet to be developed, to any extent, in this country. Narrow conceptions will continue to prevail just so long as we measure our dental education with the yardstick of finance.

Hundreds of young men will, in the coming weeks, pass out from the dental colleges of this country into the greater world of dentistry, and that after three years of study and practice. Are the teachers satisfied with the result? The mason, the printer, the blacksmith, the carpenter, the jeweller, all require from the novitiate the earnest work of, at least, five years, but dentistry, that combines the science that belongs to and is part of medicine, with the skill equally a part of the mechanician, is supposed to be acquired in three years of from seven to nine months each! The farcical character of this must be plain to the average intellect, and requires no argument to demonstrate its weakness, yet the forces in control in this country have placed the stamp of regularity upon this period of probation, and have forced all the dental colleges in this country to adopt this as the true solution of the educational problem. It was, and will continue to remain, the sacrifice upon the altar of financial expediency, a sacrifice that should bring the blush of shame to every dental educator in this land. With the curriculi of the schools increased beyond the conception of the dental educators of 1850, the length of time has been extended but one year and the length of sessions increased only three to four months.

While the outcome has been, on the average, better than could have been expected from such a policy, it is beyond question that the harvests ingathered from the dental colleges have not equalled expectations, and it is equally certain that those secured in the coming years, under the same policy, will not redound to the honor of these institutions. The graduates of to-day will without doubt carry dentistry to a much higher position in the future than it occupies at present, but this will not be due to the college training, as much as it will be to college inspiration. The men who made dentistry in the past, and developed it to its present standing, did not gather the knowledge necessary to accomplish this through college training, but rather to personal application and years of earnest work. While it is true, therefore, that all colleges, of whatever kind, simply furnish the means and inspiration to work, it must be conceded that the more distributed this is in proper fields of professional labor, the more valuable will be the outcome to the individual.

The truth of this must be manifest to any one taking a broad view of dental literature the world over. It has been stated that the one idea that medical training is the one safe road to dental education seems to prevail universally in Continental Europe. The proof of this is to be found in the dental literature of the various countries. The science of dentistry will be developed there to the practical exclusion of its mechanical side. There is still in this country a combination of the practical and scientific, with a predominance of the former, but the tendency is toward the latter, and a marked deterioration of ability in the direction of the former.

Can any one foretell the quality of the dental harvest of the future? Unless there be a decided change in the educational methods of the dental world at large, the product of the dental colleges of the future will be a hybrid that will fail to be worthy to co-operate with science, on the one hand, or the mechanism of skilled labor on the other. Will the dental educators of this country consider the situation seriously?

